# Correction: Integrative analysis of genomic variants reveals new associations of candidate haploinsufficient genes with congenital heart disease

**DOI:** 10.1371/journal.pgen.1009809

**Published:** 2021-09-21

**Authors:** Enrique Audain, Anna Wilsdon, Jeroen Breckpot, Jose M. G. Izarzugaza, Tomas W. Fitzgerald, Anne-Karin Kahlert, Alejandro Sifrim, Florian Wünnemann, Yasset Perez-Riverol, Hashim Abdul-Khaliq, Mads Bak, Anne S. Bassett, D. Woodrow Benson, Felix Berger, Ingo Daehnert, Koenraad Devriendt, Sven Dittrich, Piers EF Daubeney, Vidu Garg, Karl Hackmann, Kirstin Hoff, Philipp Hofmann, Gregor Dombrowsky, Thomas Pickardt, Ulrike Bauer, Bernard D. Keavney, Sabine Klaassen, Hans-Heiner Kramer, Christian R. Marshall, Dianna M. Milewicz, Scott Lemaire, Joseph S. Coselli, Michael E. Mitchell, Aoy Tomita-Mitchell, Siddharth K. Prakash, Karl Stamm, Alexandre F. R. Stewart, Candice K. Silversides, Reiner Siebert, Brigitte Stiller, Jill A. Rosenfeld, Inga Vater, Alex V. Postma, Almuth Caliebe, J. David Brook, Gregor Andelfinger, Matthew E. Hurles, Bernard Thienpont, Lars Allan Larsen, Marc-Phillip Hitz

The thirteenth author’s name is spelled incorrectly. The correct name is: D. Woodrow Benson.
