# Supplementary figures and images for: Integrative analysis of genomic variants reveals new associations of candidate haploinsufficient genes with congenital heart disease
Source: PLoS Genet. 2021 Jul 29;17(7):e1009679. doi: 10.1371/journal.pgen.1009679 (PMC8354477; doi:10.1371/journal.pgen.1009679)

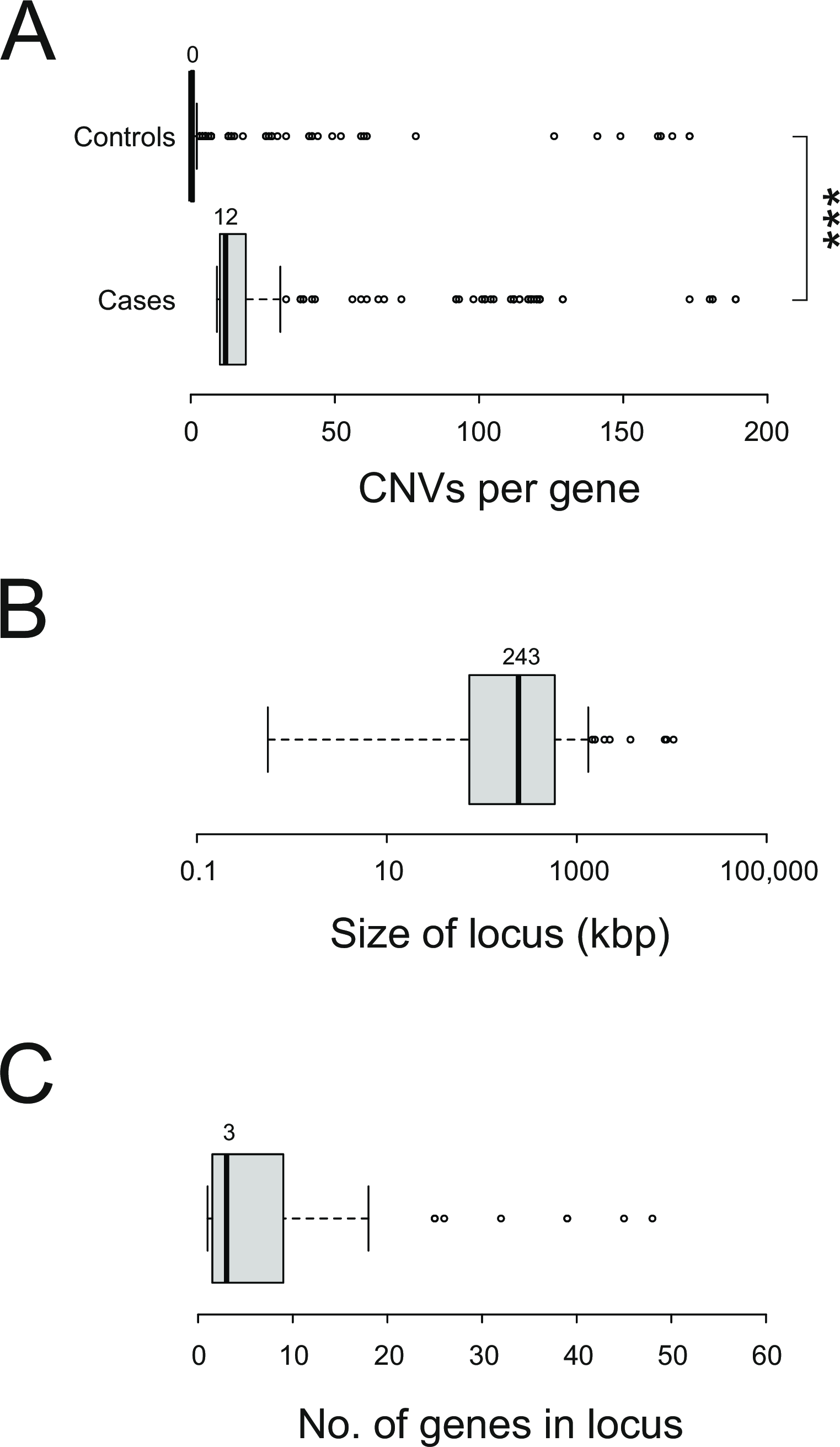

Supplement: S1 Fig — A) CNVs per gene. Overlapping CNVs for each of the 528 significant candidate genes are shown as box-and-whiskers plots. Statistically significant difference was observed between the two distributions (Mann-Whitney test, ***: P<0.001). B) Size of loci in kilobase-pairs (kbp). C) Number of genes per locus. Median values are shown above each box. (TIF) [file pgen.1009679.s001.tif]

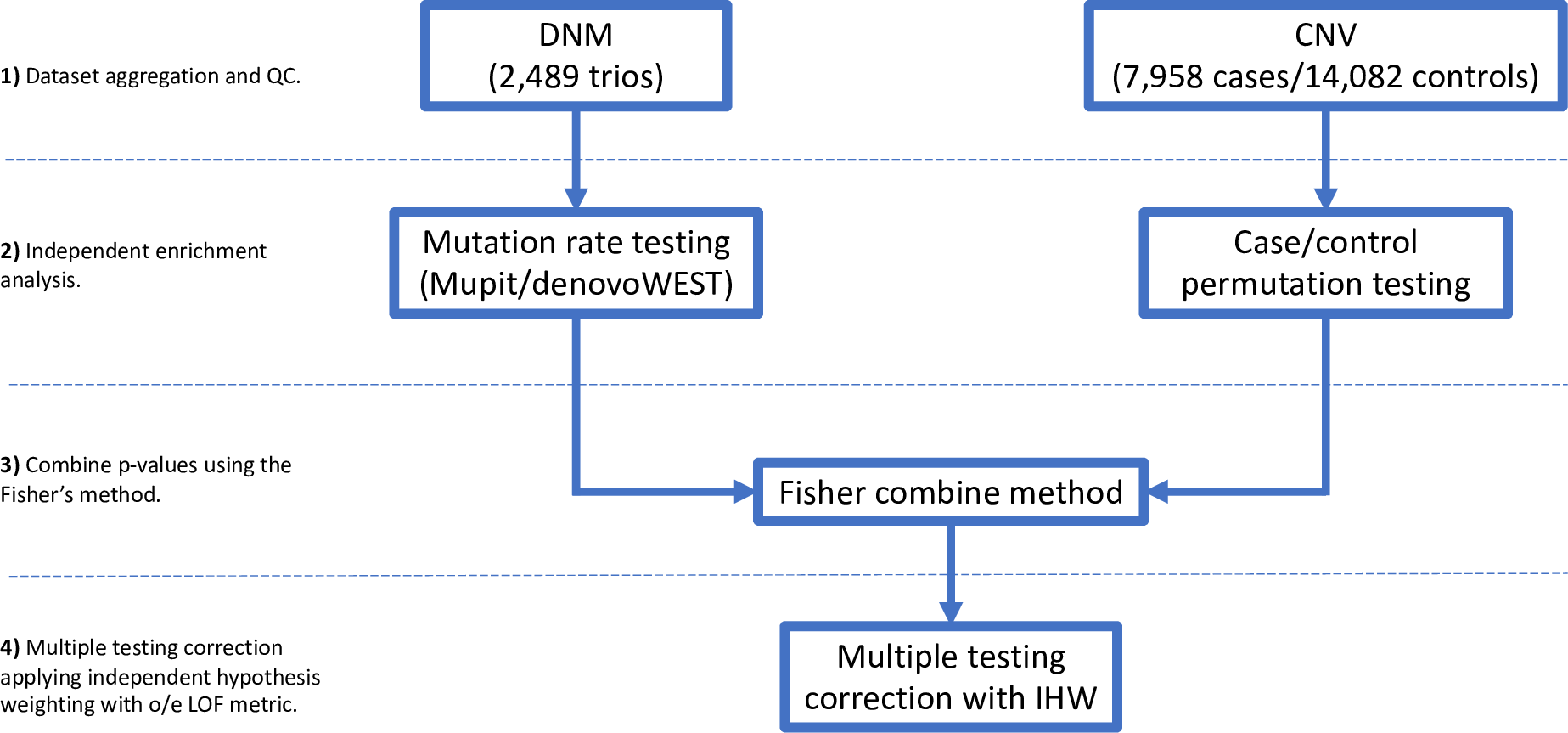

Supplement: S2 Fig — The workflow follows four major steps: 1) Data aggregation and quality control of both DNV data and CNV data, 2) DNV rate-based enrichment testing and CNV deletions case/control association analysis at gene level are performed independently, 3) the results are combined using the Fisher method and 4) P-values are Bonferroni corrected using the Independent Hypothesis Weighting method (IHW). As independent covariate for the IHW method, the o/e LOF ratio upper bound fraction (LOEUF) was used. (TIF) [file pgen.1009679.s002.tif]

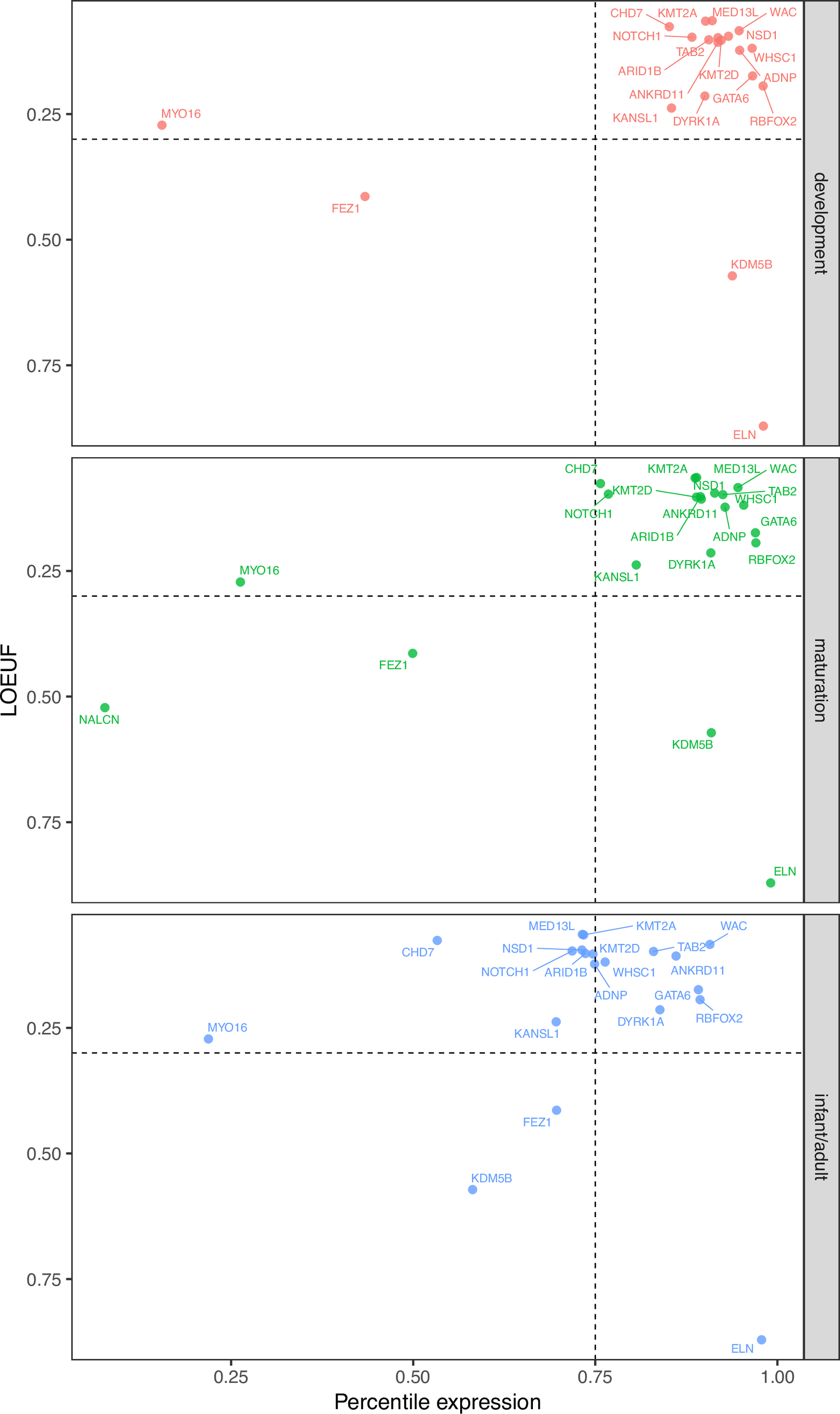

Supplement: S3 Fig — Panels show three different heart development stages: early development (red), maturation (green) and infant/adult (blue). The x-axis denotes the percentile rank of heart expression in the heart. The y-axis denotes the o/e LOF ratio upper bound fraction (LOEUF) from gnomAD. Dashed lines denote the threshold for highly expressed genes (expression rank > = 0.75) and highly LOF constrained genes (LOEUF < = 0.30). (TIF) [file pgen.1009679.s003.tif]

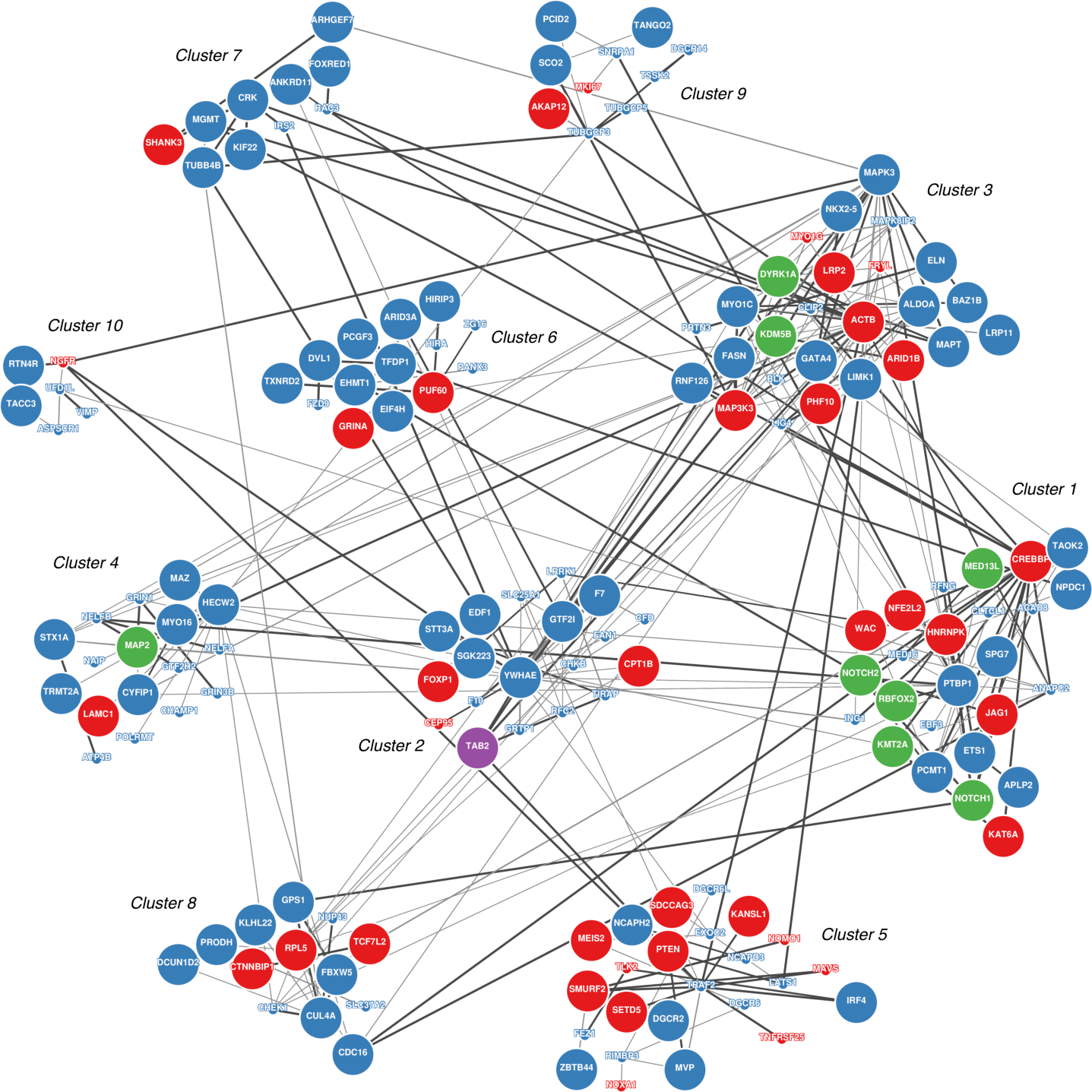

Supplement: S4 Fig — Ten sub-clusters were identified using GeNets. Proteins are shown as nodes, interactions as edges. Enrichment for CNVs (blue), DNVs (green) or both independently (purple) are highlighted. Proteins with no specific enrichment for CNV and/or DNVs but with B-H adjusted metaP < 0.05 are highlighted in red. The size of the circles denotes if the gene was found significantly highly and/or differentially expressed in the heart (large circles: significant expression; small circles: non-significant). (TIF) [file pgen.1009679.s004.tif]

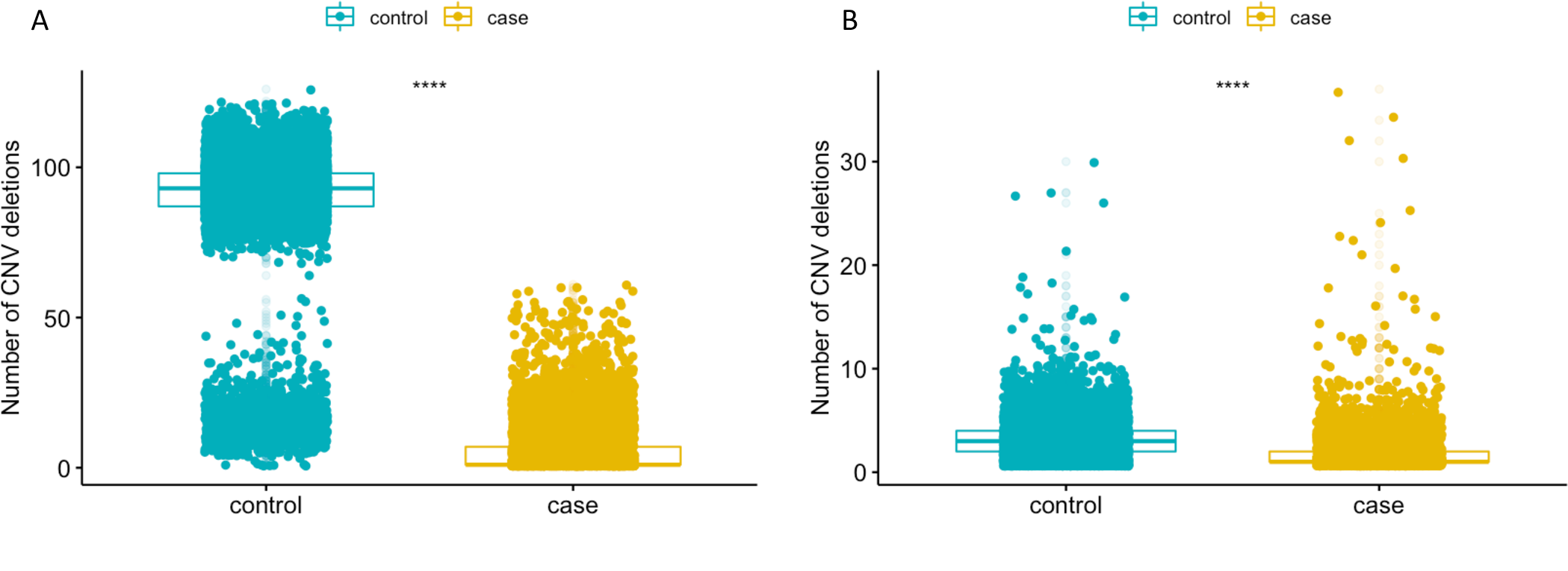

Supplement: S5 Fig — Distribution of the number of CNV deletions per individual in both control and CHD case cohorts before (A) and after (B) applying the quality control filtering approach. Differences between the distributions were tested using a two-sided Wilcoxon rank sum test. ****: P<0.0001. (TIF) [file pgen.1009679.s005.tif]
