# Supplementary material for: Integrative analysis of genomic variants reveals new associations of candidate haploinsufficient genes with congenital heart disease
Source: PLoS Genet. 2021 Jul 29;17(7):e1009679. doi: 10.1371/journal.pgen.1009679 (PMC8354477; doi:10.1371/journal.pgen.1009679)
Supplement: Attachment — Submitted filename: Response_to_Reviewers_PGEN.docx [file pgen.1009679.s021.docx]

**Response to the reviewers**

Firstly, we would like to thank the reviewers for their detailed review and suggestions to improve the current version of the manuscript. We have completed significant revisions based on the reviewers’ comments, which has considerably improved the manuscript.

**Comments to the Authors:**

**Reviewer #1:**

This paper by Audain et al is a massive study collecting CNVs/DNVs data across multiple sources in an attempt to increase the power to determine involvement in CHD, and then explore these CNVs and DNVs to find individual genes and networks. They focused only on solidly pathogenic changes due to the nature of the case data, a trade off to using all CNVs that could identify new CNVs worth consideration as novel pathogenic variants. A large control group was selected across several datasets. The authors have used orthogonal analyses for several parts of the study (such as using Mupit and DeNovoWEST) when no one gold standard exists, and then considered the genes in the CNVs in different ways (straight association, as part of heart tissue expression, and then as differential expression, and as a network analysis). Overall, the study is quite well executed, and the paper provides nice confirmation of known information (specific CNVs, known genes and involvement of specific pathways). In addition, they identify several novel genes that warrant investigation.

1. The methods are scant in the description of the study design.

R/ We thank the reviewer for pointing out this issue. The description of the methods has been moved and adapted from the supplemental information (added from page 21-26 in the main manuscript). We agree this will help readers to better understand the workflows implemented in our study.

2. The authors should state their search strategy for the literature and the public datasets chosen. Of the studies identified, how were they selected for inclusion or exclusion?

R/ We tried to focus on Caucasian samples since most studies have been performed in this population, to decrease the impact of population effects. However, we were not able to access primary data, so we cannot exclude the possibility that non-Caucasian samples were also included. We included both non-syndromic and syndromic CHD cases. Samples for the CNV meta-analysis were obtained by contacting CNV registries and authors from published/unpublished studies or downloaded/extracted available CNV calls from public databases and PubMed publications. We sampled all available and accessible studies as of February 2018 cited in PubMed. Samples were mapped to the human genome build NCBI37/hg19 (see below, question 3). Supplemental Tables 1-3 provide a description of the cohorts and studies accessed and the number of samples incorporated.

A description of the data sampling strategy has been added to the Materials and Methods section (paragraph 2 - page 21, changes highlighted in yellow)

3. Were the CNV boundaries determined using the same build of the genome?

R/ We have clarified this in the materials/methods section of the manuscript (paragraph 2 - page 22, changes highlighted in yellow) as follows:

All the CNV boundaries were determined using genome build NCBI37/hg19. For the CNVs provided in hg18, we used the Assembly Converter (https://www.ensembl.org/Homo_sapiens/Tools/AssemblyConverter) build on CrossMap (http://crossmap.sourceforge.net/) to convert samples to NCBI37/hg19 (PMID:24351709). Also, an extra validation step of all CNV boundaries was performed using the R-package *BSgenome.Hsapiens.UCSC.hg19 (DOI: 10.18129/B9.bioc.BSgenome.Hsapiens.UCSC.hg19)*

4. How did the authors determine the controls and cases were of similar ancestral groups? While this is likely less of a problem for CNVs, it would be helpful to note any differences between groups.

R/ As described in answer to question 2, most of the CNV cases and controls were collected from Caucasian cohorts. Because we could only access primarily summary data rather than primary raw data, a direct comparison to assess potential differences in ancestry was not possible using the current dataset. Nevertheless, we tried where possible to include matched-ancestry studies and small case series, but we cannot exclude the possibility that non-Caucasian samples may have been included in the study.

As stated in question 2, we have added a more detailed cohort description in the paper's material/methods section.

5. Did the authors include all genes within a CNV, or was there some selection?

R/ We want to thank the reviewer for this question. A total of 19,969 human protein-coding genes were analysed in the present study. These genes were accessed through the GENCODE database version 19 (https://www.gencodegenes.org). When a CNV spanned two or more genes, all affected protein-coding genes were considered in the analysis.

We have added a more detailed description of the gene selection process in the manuscript (paragraph 2 - page 8, changes highlighted in yellow).

6. A definition of the different gene sets used in the analysis is needed. For example, it is not clear what difference there is between monoallelic and non-syndromic sets.

R/ Thank you for bringing this to our attention. We have added a paragraph in the materials and methods section of the paper to provide sufficient additional information regarding the selection of the used gene sets (paragraph 1 - page 22, changes highlighted in yellow). Also, we would like to highlight that the gene sets are listed in Supplementary Table S13.

7. Was the phenotype information clean enough and were there enough individuals to explore the CHDs by subgroups? The genetic architecture is quite different for instance between conotruncal defects and left sided defects.

R/ Phenotype information was not consistent across the data collected as part of the meta-analysis, as different groups provided phenotypes. Two clinicians reviewed all phenotypes separately, to ensure consistency and accuracy. Both Human Phenotype Ontology (PMID:33264411) and International Paediatric and Congenital Cardiac Codes (PMID:29286277) were applied. Cardiac lesions were annotated with a specific and more general classification to allow combination of related phenotypes, without loss of specific cardiac details. Given the nature of a meta-analysis, precise phenotyping is complex and should be done in a more gene-based context. Future studies could be improved by using more consistent phenotyping and for example integrating HPO-Terminology or other code-based systems (Fyler and IPCC codes).

Following the reviewer’s suggestion, we explored two major sub-groups (Conotruncal malformations and LVOTO) at both CNV and DNV level (Table 1 summarises the number of affected probands per category). We performed a combined CNV/DNV meta-analysis, stratified by CHD sub-category. Given the heterogeneous dataset, we focused the sub analysis on cases with a clear phenotype description and excluded complex malformations and cases with uncertain phenotype descriptions.

Table 2 shows the four genes surviving the Bonferroni correction (*adjusted metaP < 0.05*) and the sub-phenotypes in which they were found to be enriched. Although such analysis can reveal the distinct genetic architecture between different CHD sub-types, the small numbers in Table 2 reinforce the need for larger datasets to increase the power to detect significant genes at a sub-phenotype level.

We have added two paragraphs in the current manuscripts explaining these results (paragraph 3 - page 11 and paragraph 3 - page 17, changes highlighted in yellow). We also amended the cohort description on page 21 marked in yellow.

Table 1. Number of affected probands per CHD sub-type, by corresponding data set.

|  | Probands | |
| --- | --- | --- |
|  | **DNV** | **CNV** |
| **Conotruncal** | 234 | 873 |
| **LVOTO** | 351 | 594 |

Table 2. Meta-analysis of CNV/DNV stratified by CHD sub-type (Conotruncal and LVOTO), showing four genes with Bonferroni corrected metaP < 0.05.

|  | **CNV** | | | **DNV** | | **Combined** | |  |
| --- | --- | --- | --- | --- | --- | --- | --- | --- |
| **gene** | **cases** | **control** | **p_cnv** | **nsDNV** | **p_dnv** | **metaP** | **adj metaP** | **sub-phenotype** |
| *KMT2D* | 0 | 0 | 1 | 8 | 2.40E-17 | 9.4E-16 | 3.6E-12 | lvoto |
| *KMT2A* | 0 | 0 | 1 | 4 | 2.08E-12 | 5.8E-11 | 2.2E-07 | lvoto |
| *TAB2* | 1 | 0 | 0.04 | 2 | 2.86E-06 | 1.9E-06 | 7.2E-03 | lvoto |
| *NSD1* | 0 | 1 | 1 | 3 | 2.76E-08 | 5.1E-07 | 9.8E-03 | conotruncal |

This table has been added as Supplemental Table 11.

8. The authors should also acknowledge an additional bias of the case data collected. The CNVs are highly selected from only the cases published due the presence of a CNV so are not representative of all individuals with CHD.

R/ We agree with the reviewer and are aware of this bias. This is discussed on page 19 to ensure the reader is aware of this problem (paragraph 2, highlighted in yellow).

**Reviewer #2:**

In this manuscript, the authors present their study on an integrative analysis to identify copy number variants (CNVs) and de novo intragenic variants (DNVs) in patients with congenital heart disease (CHD) with sporadic thoracic aortic aneurysm. The authors used published and publicly available datasets of CNVs and DNVs identified in probands with CHD (10,447) to perform a genome-wide integrative meta-analysis. To achieve their goal the authors implemented a statistical approach which allows the integration of different types of genomic variants. Based on their study they identified 21 significant haploinsufficient CHD genes. Among these genes 14 are already known as CHD genes and 7 have not previously been associated with CHD. The authors evaluated expression pattern of these genes in the heart using available data from the recent human atlas study. In summary, this study used astutely available datasets to identify novel CHD genes.
Although the basic insight regarding the identification of novel genes involved in CHD is very interesting, there are some weaknesses in the data reported in this study.

Major concerns:

1. My main concern is related to diversity of CHD used in this study. The authors used CHD as a generic denomination. There is no detail regarding the type and class of CHD used in this study. For instance, conotruncal and non-conotruncal groups would be a good indicator to better classify these CHDs in order to improve the analysis.

R/ We agree that uncertainties around the diagnosis of specific CHD type are a potential weakness of the study. We hoped to ameliorate this risk by using a large dataset to compensate. We have performed a sub-analysis of the data based on specific groups of heart malformations (see our answer to reviewer #1, Q7). The data has been added to the results section (paragraph 2 - page 11 and paragraph 3 - page 17/18, changes highlighted in yellow).

2. I wonder if there is a bias in using these publicly available datasets since their integrative analysis revealed significant enrichment for the known CHD genes, which is mainly explained by the contribution of monoallelic CHD genes and syndromic CHD gene set. This should be better discussed and the authors should propose alternative analysis to identify non-syndromic CHD genes.

R/ We agree that there is the possibility that our analysis is biased towards known monoallelic and syndromic genes. A likely reason for such a bias is that our meta-analysis utilised previously published datasets, which had helped to identify or strengthen the initial association. We were aware of this bias and the aim of this study was to find additional genes which had not reached significance previously. We have added and reworded the discussion on page 20, paragraph 1 (highlighted in yellow) highlighting this aspect to the reader. Also, we have discussed in more detail future strategies to improve CHD gene and network discovery. Important requirements should include more detailed phenotypic description, access and sharing of primary data to improve associations for syndromic and non-syndromic CHD patients (page 20, paragraph 2 marked in yellow).

3. It is very surprising to see that one of the most frequent syndromes (22q11/DiGeorge syndrome) is not among the CNV deletion syndrome regions identified in this study. Particularly, the authors mentioned that the distribution of CNVs that overlap known microdeletion syndrome such as DiGeorge syndrome is overrepresented in the dataset. I wonder about the quality of the analysis and the filters applied to identify CHD genes.

R/ We want to thank the reviewer for highlighting this issue. We have observed a distinct pattern in the distribution of rare CNV deletions within the 22q11 locus (Figure 1). We attributed the non-enrichment of this region to the presence of multiple genes with a high number of CNV-controls. However, known CHD genes such as *TBX1* and *TANGO2* were significantly enriched for CNV-cases (see Supplemental Table 6). This is shown below for the 22q11.2 region.

Figure 1. Distribution of rare CNV deletions (MAF 0.01) on chromosome 22. The locus 22q11.21 shows a distinct distribution pattern among CNV cases (blue) compared to CNV controls (red). Genes within 22q11.21 with significant enrichment from case/control permutation testing are labelled in blue, non-significant genes are labelled in red. Supplemental Table 6 (manuscript) shows the case/control counts and p-value from permutation testing for every gene.

4. The authors used bulk-RNAseq dataset to determine if the 21 significant genes are expressed in the heart at different developmental time points. However, there is no information regarding the region of the heart where these genes are expressed. This information could be useful in relation to the type of CHD they are associated with.

R/ We agree with the reviewer on the importance of integrating and knowing the sampling location of the anatomic heart region to improve the gene discovery analysis. Unfortunately, the work published by Cardoso *et al.* (<https://doi.org/10.1038/s41586-019-1338-5>) does not include detailed information about which part of the heart was analysed at different development stages (Methods and Supplemental Table 1 and 2). Nevertheless, this data was, in our opinion, the most complete temporal human transcriptomics atlas publicly available. It covers early to late cardiogenesis processed on the same platform to ensure minimal technical and biological biases. Therefore, it was not possible to establish a link between significantly expressed genes in different heart regions and specific types of CHD.

Minor points:

1. S6 Table: It is not evident to find the 528 genes. The authors should better highlight them).

R/ We have added an extra column (is_significant) to mark those genes (n=528) with adjusted p-value < 0.05. The new column has been described in the corresponding Table S6 information sheet.

2. The authors should better explain why it is important to known that genes remain highly expressed in adulthood.

R/ We agree that this information is not important for the analysis, and we have deleted this part of the sentence.

3. It is not clear why the authors have conducted a systems-level analysis to identify global mechanisms if there is no mechanism proposed in this study.

R/ The mechanisms suggested from the systems-level analysis is discussed on pages 18 and 19.

**Reviewer #3:**

The manuscript by Audain et al. performs a meta-analysis of copy number variants and de novo variants identified by exome sequencing from previously published studies. The case and control cohort sizes are large and the authors have established an extensive cohort. Using burden analyses the authors identified 21 genes associated with CHD including 14 previously known genes and 7 “novel” genes. In many ways this manuscript provides proof of principle that meta-analyses from a large number of studies, including single case studies, can be performed and platform results can be harmonized for both DNV and CNVs and this is an important contribution. However, the novel findings are limited and the goal of using integrative analyses to identify CHD disease genes in multifactorial inheritance is not accomplished here but it does provide a path forward. It would improve the impact of the manuscript to apply the expression and/or network analyses to a somewhat broader group of candidate genes.

Major comments:

1. The study focuses on CNV deletions but it would be useful to perform analyses for duplications as well particularly for overlaps with the deletions as this would further support dosage sensitivity and mechanisms.

R/ We agree that this an interesting analysis. We have therefore performed CNV analysis based on rare CNV-duplications and found 19 genes with significant burden of CNV-duplications in cases which also were significant in the CNV-deletion analysis (Table 3). Unfortunately, none of the genes with significant enrichment was significant in our combined analysis with evidence from DNV and CNV deletions side, which would have warranted further investigation in the current study. We think that given our paper was looking predominantly at haploinsufficiency genes, the analysis performed would not add to the current manuscript and considered to leave it out.

Table 3. Gene-based case/control CNV-duplications permutation testing results (PLINK). case: number of CNV-dup cases, control: number of CNV-dup controls, p_value: P-value from permutation testing, p_value_adj: Bonferroni corrected P-value from permutation testing.

| **gene** | **case** | **control** | **p_value** | **p_value_adj** |
| --- | --- | --- | --- | --- |
| *AMY1C* | 28 | 8 | 1.00E-04 | 7.00E-04 |
| *APOD* | 16 | 1 | 1.00E-04 | 1.20E-03 |
| *ARHGEF35* | 18 | 6 | 1.00E-04 | 4.80E-02 |
| *AURKAIP1* | 13 | 1 | 1.00E-04 | 1.52E-02 |
| *CNTNAP3B* | 116 | 45 | 1.00E-04 | 1.00E-04 |
| *CPSF3L* | 17 | 3 | 1.00E-04 | 5.50E-03 |
| *CPTP* | 17 | 3 | 1.00E-04 | 5.50E-03 |
| *GTF2H2* | 70 | 29 | 1.00E-04 | 1.00E-04 |
| *LINC00116* | 32 | 4 | 1.00E-04 | 1.00E-04 |
| *MALL* | 38 | 4 | 1.00E-04 | 1.00E-04 |
| *MUC20* | 89 | 1 | 1.00E-04 | 1.00E-04 |
| *MUC4* | 49 | 1 | 1.00E-04 | 1.00E-04 |
| *NAIP* | 81 | 37 | 1.00E-04 | 1.00E-04 |
| *NEIL2* | 10 | 0 | 2.00E-04 | 3.03E-02 |
| *NPHP1* | 38 | 4 | 1.00E-04 | 1.00E-04 |
| *PUSL1* | 15 | 3 | 1.00E-04 | 2.66E-02 |
| *SCNN1D* | 13 | 2 | 1.00E-04 | 4.38E-02 |
| *SPATA31A6* | 65 | 40 | 1.00E-04 | 1.00E-04 |
| *TAS1R3* | 17 | 3 | 1.00E-04 | 5.50E-03 |

2. The control population is not described well. How carefully phenotyped are these controls?

The description of the control data was indeed scant, and we thank the reviewer for pointing this out. The controls used were genotyped in the context of common disease and analysed at the Wellcome Trust Sanger Institute. The majority of controls has been part of the Wellcome Trust Case Control Consortium 2, (WTCC2, Link: <https://www.wtccc.org.uk/ccc2/wtccc2_studies.html>) and the Genetic Association Information Network (GAIN, Link:<https://fnih.org/what-we-do/major-completed-programs/genetic-association-information-network>). In addition, we also accessed a previously published cohort by the Ottawa Heart Institute cohort in patients with myocardial infarction (PMID:19371834). We can not rule out the possibility that some of these samples have CHD, but the majority of these have been sampled as adults in the context of common disease, without known evidence of CHD.

We therefore added a short paragraph in the material and methods section (paragraph 2 – page 21) and added additional info to Supplemental Table 3, which links to more detailed information regarding the control data.

3. Please clarify how genes were assigned to syndromic, non-syndromic, monoallelic, biallelic gene sets and other gene sets.

What proportion of the genes are shared between lists and how is this accounted for in analyses?

R/ We want to thank the reviewer for suggesting that this part needs clarification. The derivation of the lists is addressed above in response to question 6 from Reviewer 1. CHD genes were stratified by syndromic status (syndromic / non-syndromic) and by allelic requirement (mono-allelic / bi-allelic). There is no overlap between the CHD associated genes within a category (e.g. syndromic vs non-syndromic or mono-allelic vs bi-allelic). However, overlap is expected among the syndromic status and allele requirement genes, since one is phenotype-oriented and the other mechanism-oriented. This overlap is evident, for example, among syndromic and mono-allelic CHD genes (Table 4). In addition, we expect overlap between CHD genes and genes classified as DD and HI. Table 4 shows the pairwise proportional overlaps for the different gene list. Also, we have formatted the manuscript’s Supplemental Table 13 (Gene sets) to make it easier to explore the shared genes among the different sets. The gene set enrichment analysis was done independently for every set.

Table 4. Pairwise comparison between different gene lists. n_overlaps denotes the number of genes shared between sets. *overlap_prop = intersect (s1, s2) / union (s1, s2) * 100. AR: autosomal recessive genes, HI: Haploinsufficient genes.

| Set 1 | Set 2 | n_overlaps | *overlap_prop (%) |
| --- | --- | --- | --- |
| syndromic_chd | nonsyndromic_chd | 0 | 0 |
| syndromic_chd | monoallelic_chd | 101 | 40.56 |
| syndromic_chd | biallelic_chd | 92 | 37.86 |
| syndromic_chd | developmental_disorder | 79 | 16.7 |
| syndromic_chd | AR | 61 | 4.55 |
| syndromic_chd | HI | 63 | 13.97 |
| nonsyndromic_chd | monoallelic_chd | 29 | 18.24 |
| nonsyndromic_chd | biallelic_chd | 23 | 15.33 |
| nonsyndromic_chd | developmental_disorder | 6 | 1.56 |
| nonsyndromic_chd | AR | 7 | 0.57 |
| nonsyndromic_chd | HI | 4 | 1.15 |
| monoallelic_chd | biallelic_chd | 0 | 0 |
| monoallelic_chd | developmental_disorder | 79 | 20.63 |
| monoallelic_chd | AR | 11 | 0.84 |
| monoallelic_chd | HI | 49 | 13.07 |
| biallelic_chd | developmental_disorder | 3 | 0.68 |
| biallelic_chd | AR | 55 | 4.42 |
| biallelic_chd | HI | 1 | 0.25 |
| developmental_disorder | AR | 24 | 1.61 |
| developmental_disorder | HI | 122 | 24.21 |
| AR | HI | 16 | 1.1 |

4. Previous studies from some of the authors of this study have demonstrated enrichment of CNVs in specific subphenotypes of CHD. Additional information and analyses performed on subphenotypes would be useful to include as well as whether any previous findings can be expanded.

R/ We thanks to the reviewer for this suggestion. This issue has been addressed in response to reviewer 1, question 7.

5. For TAA, previous studies from some of the authors indicate a higher burden of CNVs in familial TAA. What is the representation of familial vs sporadic in this meta-analysis and can any additional comments be made about CNV burden?

R/ The majority of cases with TAA have been published in a previous paper. In the present work, the majority of cases included are sporadic TAA (Prakash et al. 2010, PMID:21092924)

6. In Table 7 the method for identifying CNVs in controls needs explanation or better descriptors. Why are the CNV counts so high in well described genomic disorders such as 22q11.2/VCFS/DiGeorge (986 controls), 1p36 deletion syndrome (886 controls) or Cri du Chat (1488 controls). These values should also have established a MAF > .01 prompting filtering. More broadly, it is surprising that TBX1 was not identified in the burden analyses. Could the authors comment particularly as it was noted in the discussion that 22q11.2 cases were overrepresented and so TBX1 seemingly should have shown significant association by burden testing.

We agree that this could have been better explained. We have added the explanation in the Materials and Methods section to explain the test for known deletion syndromes (paragraph 1 - page 24, highlighted in yellow). All CNVs deletion passing the QC filtering step were considered for the case/control burden analysis. As pointed out in the response to reviewer #2 (Q3), we have observed a distinct pattern in the distribution of rare (MAF 0.01) CNV deletions in cases and controls within 22q11.2 locus (Figure 1). This can lead to an excess of controls when considering the complete region in the permutation-based burden test. However, looking at individual genes, we have observed that well-established genes associated with genomic disorders are strongly enriched in CNV-cases compared to controls (see Supplemental Table 6, e.g. *TBX1* and *TANGO2*). For example, *TBX1* is significant in the CNV burden test (P = 1e-04, 120 case-CNVs, 13 control-CNVs), but *TBX1* was not significant in the DNV test.

Figure 2 shows the distribution of CNV-cases and CNV-controls within 1p36 and Cri du Cha regions. For 1p36, we observed some genes at the start and end of the region overlapped for an elevated number of CNV-controls. Similarly, in the Cri du Chat syndrome deletion region, the majority of controls were observed in the genes *ZDHHC11B, ZDHHC11* and *BRD9*. None of the other genes associated with Cri du Chat syndrome in our cohort did reach significance (Supplemental Table 6, chr5:10001-chr5:12533304).

It is also worth to note that some well-established deletion syndromes such as 22q11.2 show variable clinical findings, which might explain occurrences in the control datasets.

Figure 2. Distribution of rare CNV deletions (MAF 0.01) on two known deletion syndrome regions: a) 1p36 and b) Cri du Cha. Plot shows the distribution of CNV-cases (blue) and CNV-controls (red). The case/control counts and p-value from permutation testing for every single gene within the regions can be accessed in the Supplemental Table 6 (manuscript).

7. Some of the “novel” genes cause syndromes that do have well described CHD such as Wolf-Hirschhorn or ARID1B. The manuscript could be improved by a better discussion of modifiers or oligogenic mechanisms and reduced penetrance of CHD as the rule even in genetic syndromic conditions.

Thank you for this comment. Variable expressivity and reduced penetrance are prevalent in both syndromic and non-syndromic CHD as the reviewer has pointed out. We have improved the discussion about this issue (paragraph 2 – page 20, highlighted in yellow).

8. The data on TAA do not contribute significantly to the manuscript.

Whilst the TAA cohort is much smaller than the CHD cohort, we feel it is an important phenotype to explore given there is an established overlap in genes causing CHD and TAA. This includes duplication of MYH11 (PMID:21698135), HEY2 (PMID:32820247), ABL1 (PMID:[28288113](https://www.ncbi.nlm.nih.gov/pubmed/28288113)), FBN2 (PMID:28991257, PMID:27479907) and SMAD3 (PMID:21217753, PMID:[24711937](https://www.ncbi.nlm.nih.gov/pubmed/24711937)). Whilst the negative finding may not contribute as important a result as a novel CHD gene here, we feel it is important to highlight and explore this link.

Because of this emerging overlap of genes causing both CHD and TAA we feel it is important to include the TAA cohort to explore this relationship further, even if it has not yielded any significant genes in this instance. We have included the comment that this may be as a result of the small sample size of individuals with TAA.

9. The manuscript is difficult to follow from an analysis standpoint at times. It would benefit from a flow diagram of analyses and/or including more of the methods within the manuscript.

R/ Thank you for this suggestion. We have moved the methods (see question one reviewer one) section from the supplement into the main manuscript. We agree this will make it easier for the reader to follow.

Minor:

1. The size cutoff of 5 kb and 20 Mb is relevant and should be moved to the methods of the main manuscript.

R/ This information has been included in the main manuscript within the methods section.

2. Fig. S4 – what does purple denote?

R/ The purple denotes those genes which are enriched for both CNVs and DNVs. It has been highlighted in the corresponding legend description.

3. Fig. 5 - the x axis (Score) needs an explanation in the legend

R/ We have included the explanation of the x-axis (Score) in the corresponding figure legend.

4. Heading p. 12 CNV/DNVs hinder the function of specific protein complexes should be reworded since this has not been proven by this study.

R/ The heading has been reworded to “CNV/DNVs burden of specific protein complexes” (page 13).

**Have all data underlying the figures and results presented in the manuscript been provided?**
Large-scale datasets should be made available via a public repository as described in the *PLOS Genetics* [data availability policy](http://journals.plos.org/plosgenetics/s/data-availability), and numerical data that underlies graphs or summary statistics should be provided in spreadsheet form as supporting information.

Reviewer #1: Yes

Reviewer #2: Yes

Reviewer #3: Yes

PLOS authors have the option to publish the peer review history of their article ([what does this mean?](https://journals.plos.org/plosgenetics/s/editorial-and-peer-review-process#loc-peer-review-history)). If published, this will include your full peer review and any attached files.


If you choose “no”, your identity will remain anonymous but your review may still be made public.


**Do you want your identity to be public for this peer review?** For information about this choice, including consent withdrawal, please see our [Privacy Policy](https://www.plos.org/privacy-policy).

Reviewer #1: **Yes:** Kim L McBride

Reviewer #2: No

Reviewer #3: No
