# Supplementary material for: Integrative analysis of genomic variants reveals new associations of candidate haploinsufficient genes with congenital heart disease
Source: PLoS Genet. 2021 Jul 29;17(7):e1009679. doi: 10.1371/journal.pgen.1009679 (PMC8354477; doi:10.1371/journal.pgen.1009679)
Supplement: Attachment — Submitted filename: Response_to_Reviewers_PGEN.docx [file pgen.1009679.s022.docx]

**Response to the reviewers**

We would like to thank the reviewers for their constructive feedback. We have completed a minor revision based on the reviewers’ comments, which has improved the current version of the manuscript.

**Comments to the Authors:**

**Reviewer #2:**

The manuscript by Audain et al. has been well revised. The authors have completed significant revisions based on my comments. I find the current version of the manuscript considerably improved. The authors have replied to my main concern regarding the diagnosis of specific CHD type. They have performed a sub-analysis of the data based on specific groups of heart defects. They have also replied to my question concerning the possibly bias of their analysis since they used previously published datasets. The current version included a discussion on this specific point. The authors have satisfactorily answered my question regarding the 22q11 locus. Absence of detailed information about spatiotemporal expression of identified genes with CHD does not confirm the link these genes in the different part of the heart and the type of CHD in which they are involved. The authors should have performed a better analysis to reinforce this specific issue.

R/ We agree that a spatiotemporal analysis of the role of candidate genes during heart development could provide additional pathophysiological insight. However, we find that this would be a comprehensive addition to the current manuscript, and after consulting the editors, we have chosen not to include such analyses and experiments.

In the discussion section, we have now noted that additional analyses at single-cell resolution could strengthen disease association and provide pathophysiological information (highlighted in green, paragraph 1 - page 15).

**Reviewer #3:**

The authors have been responsive to the reviewers’ comments within the limitations of available data. The addition of the CHD phenotype subclassifications is useful and the discussion/interpretation of the findings is appropriate. The requested points for clarification have mostly been addressed. I have only minor comments.

I did not note in the original submission that CNVs were compiled for autosomes only. I would suggest the discussion/limitations make more explicit the fact that X-linked candidate genes will not be identified by the integrative approach.

R/ We thank the reviewer for highlighting this issue. We have reflected it explicitly in the manuscript as follow: “The lack of collected CNV data spanning sex chromosomes limited the analysis only to autosomal chromosomes” (highlighted in green, paragraph 2 – page 19).

The incorporation of the detailed methods is helpful. For the older platforms in the study, was the minimum CNV interval or maximum CNV interval (< 20 Mb) used? Are platform differences the explanation for the persistent differences between cases and controls even after filtering (FigS5)?

R/ All CNVs were processed applying the same QC filtering criteria, independently of the capture platform being used. This allowed us to reduce dramatically the differences in the number of CNVs between cases and controls.

Even though we observed comparable number of CNVs per individuals in the case and control set, we attributed the persistent differences to a combination of the different platforms used in the study as well as the incomplete data collected in cases compared to controls. These issues have been emphasised as a limitation of this study in the Discussion section (paragraph 2 – page 19).
